# Differentiation between bipolar disorder and major depressive disorder based on AMPA receptor distribution

**DOI:** 10.3389/fncir.2025.1624179

**Published:** 2025-08-04

**Authors:** Sakiko Tsugawa, Yuichi Kimura, Junichi Chikazoe, Hiroki Abe, Tetsu Arisawa, Mai Hatano, Waki Nakajima, Hiroyuki Uchida, Tomoyuki Miyazaki, Yuuki Takada, Akane Sano, Kotaro Nakano, Tsuyoshi Eiro, Akira Suda, Takeshi Asami, Akitoyo Hishimoto, Hideaki Tani, Nobuhiro Nagai, Teruki Koizumi, Shinichiro Nakajima, Shunya Kurokawa, Yohei Ohtani, Kie Takahashi, Yuhei Kikuchi, Taisuke Yatomi, Ryo Mitoma, Shunsuke Tamura, Shingo Baba, Osamu Togao, Yoji Hirano, Hirotaka Kosaka, Hidehiko Okazawa, Masaru Mimura, Takuya Takahashi

**Affiliations:** ^1^Department of Physiology, Graduate School of Medicine, Yokohama City University, Yokohama, Japan; ^2^Department of Neuropsychiatry, Keio University School of Medicine, Tokyo, Japan; ^3^Faculty of Informatics, Cyber Informatics Research Institute, Kindai University, Higashi-Osaka, Japan; ^4^Brain, Mind and KANSEI Sciences Research Center, Hiroshima University, Hiroshima, Japan; ^5^Radioisotope Research Center, Graduate School of Medicine, Yokohama City University, Yokohama, Japan; ^6^Center for Promotion of Research and Industry-Academic Collaboration, Yokohama City University, Yokohama, Japan; ^7^Department of Psychiatry, Graduate School of Medicine, Yokohama City University, Yokohama, Japan; ^8^Department of Psychiatry, Kobe University Graduate School of Medicine, Kobe, Japan; ^9^Department of Neuropsychiatry, Graduate School of Medical Sciences, Kyusyu University, Fukuoka, Japan; ^10^Division of Clinical Neuroscience, Department of Psychiatry, Faculty of Medicine, University of Miyazaki, Miyazaki, Japan; ^11^Department of Health Sciences, Graduate School of Medical Sciences, Kyushu University, Fukuoka, Japan; ^12^Department of Clinical Radiology, Graduate School of Medical Sciences, Kyushu University, Fukuoka, Japan; ^13^Institute of Industrial Science, The University of Tokyo, Tokyo, Japan; ^14^Department of Neuropsychiatry, School of Medical Sciences, University of Fukui, Fukui, Japan; ^15^Biomedical Imaging Research Center, University of Fukui, Fukui, Japan; ^16^The International Research Center for Neurointelligence, Institutes for Advanced Study, University of Tokyo, Tokyo, Japan

**Keywords:** AMPA receptor, bipolar disorder, depression, differentiation, machine learning

## Abstract

An accurate diagnostic method using biological indicators is critically needed for bipolar disorder (BD) and major depressive disorder (MDD). The excitatory glutamate α-amino-3-hydroxy-5-methyl-4-isoxazolepropionic acid receptor (AMPAR) is a crucial regulator of synaptic function, and its dysregulation may play a central role in the pathophysiology of psychiatric disorders. Our recently developed positron emission tomography (PET) tracer, [^11^C]K-2, enables the quantitative visualization of AMPAR distribution and is considered useful for characterizing synaptic phenotypes in patients with psychiatric disorders. This study aimed to develop a machine learning-based method to differentiate bipolar disorder from major depressive disorder using AMPAR density. Sixteen patients with BD and 27 patients with MDD, all in depressive episodes, underwent PET scans with [^11^C]K-2 and structural magnetic resonance imaging. AMPAR density was estimated using the standardized uptake value ratio from 30 to 50 min after tracer injection, normalized to whole brain radioactivity. A partial least squares model was trained to predict diagnoses based on AMPAR density, and its performance was evaluated using a leave-one-pair-out cross-validation. Significant differences in AMPAR density were observed in the parietal lobe, cerebellum, and frontal lobe, notably the dorsolateral prefrontal cortex between patients with BD and patients with MDD during a depressive episode. The model achieved an area under the curve of 0.80, sensitivity of 75.0%, and specificity of 77.8%. These findings suggest that AMPAR density measured with [^11^C]K-2 can effectively distinguish BD from MDD and may aid diagnosis, especially in patients with ambiguous symptoms or incomplete clinical presentation.

## Introduction

Mood disorders, including major depressive disorder (MDD) and bipolar disorder (BD), are complex psychiatric conditions. Approximately 10%–20% of individuals experience MDD, while 2.4% develop BD over their lifetimes (Merikangas et al., 2011; Lim et al., 2018; Gutiérrez-Rojas et al., 2020). These disorders pose significant public health challenges and remain leading causes of global disability, highlighting the substantial reduction in healthy life years attributable to these conditions (Vigo et al., 2016). BD and MDD are operationally diagnosed under the Diagnostic and Statistical Manual of Mental Disorders (DSM) (Cooper, 2018), with the primary distinction being the presence of mania in BD, absent in MDD. However, differentiating BD from MDD remains challenging, as BD often first appears with depressive symptoms and lacks early manic episodes (Hantouche et al., 2006; Smith et al., 2011; Shen et al., 2018), leading to a high misdiagnosis rate (40%–75% of patients with BD are first diagnosed as MDD) (Ghaemi et al., 1999, 2000; Hirschfeld et al., 2003; Watanabe et al., 2016; Shen et al., 2018). Moreover, BD’s episodic nature, marked by fluctuating intervals between depressive and manic/hypomanic phases, further complicates diagnosis and can delay accurate identification by 4–10 years (Ghaemi et al., 2000; Baethge et al., 2003; Hirschfeld et al., 2003; Baldessarini et al., 2007; Watanabe et al., 2016). MDD is typically treated with antidepressants, whereas BD requires mood stabilizers and second-generation antipsychotics. This difference in treatment approach necessitates the precise differentiation of BD from MDD. In patients with BD, antidepressants increase the risk of rapid cycling—frequent shifts between manic and depressive episodes—significantly heightening the likelihood of suicidal behavior (Carvalho et al., 2020). Additionally, prolonged misdiagnosis impairs quality of life and increases hospitalizations and healthcare costs (Goldberg and Ernst, 2002; Birnbaum et al., 2003; Shi et al., 2004; McCombs et al., 2007). Given these challenges, objective biomarkers are urgently needed to facilitate early and accurate differentiation between BD and MDD during depressive states. Numerous studies have explored this, including a recent one on peripheral blood-based assays, such as RNA-editing markers combined with machine learning (ML), which reportedly achieved good classification accuracy (Salvetat et al., 2024). However, these approaches often rely on systemic immunological or inflammatory signals rather than directly capturing the underlying neuronal pathophysiology. Medication use and comorbid conditions can heavily influence peripheral measurements, raising uncertainty about their reflection of neural dysfunction in BD. Consequently, a biomarker that visualizes neuronal targets in the living human brain would provide a more direct biological interpretation and reduce ambiguities inherent in blood-derived markers.

Our research focuses on the role of the excitatory glutamate α-amino-3-hydroxy-5-methyl-4-isoxazolepropionic acid receptors (AMPARs) and elucidates synaptic dysfunction in mood disorders (Hatano et al., 2024). AMPARs are the primary molecules that mediate fast transmission at glutamate synapses (Derkach et al., 2007; Jitsuki et al., 2011; Diering and Huganir, 2018; Krystal, 2020). The synaptic trafficking of AMPARs is a key molecular mechanism driving experience-dependent synaptic plasticity, which underlies fundamental cognitive processes such as learning and memory (Takahashi et al., 2003; Mitsushima et al., 2011; Takemoto et al., 2017; Abe et al., 2018; Diering and Huganir, 2018). Given their role as crucial regulators of synaptic function, AMPARs have been implicated as key molecular bottlenecks in the pathophysiology and treatment mechanisms of psychiatric disorders (Ueno et al., 2019; Yonezawa et al., 2022). Consistently, dysregulated AMPAR signaling is observed in mood disorders. In the dorsolateral prefrontal cortex, GluA2 mRNA is reduced in both MDD and BD, whereas GluA3 down-regulation appears confined to MDD (Beneyto and Meador-Woodruff, 2006). Cortico-limbic extension of this pattern is evident in the entorhinal cortex of BD, which shows concordant decreases in GluA2 and GluA3 transcripts (Beneyto et al., 2007). In rodents, pharmacological enhancement of AMPAR throughput or synaptic insertion reproduces—whereas AMPAR antagonism blocks—the rapid and sustained antidepressant-like effects of ketamine (Suzuki et al., 2023). Together with accumulating evidence that AMPAR-dependent synaptic plasticity underlies experience-driven circuit remodeling (Derkach et al., 2007; Diering and Huganir, 2018), these findings position aberrant AMPAR signaling as a mechanistically tractable pathway in mood disorders.

Extensive preclinical research has established the importance of AMPARs; however, the inability to visualize AMPARs in the living human brain limits their translation to clinical practice. To address this gap, we developed a positron emission tomography (PET) tracer, [^11^C]K-2, that can quantitatively assess AMPAR density on the cell surface *in vivo* (Miyazaki et al., 2020; Arisawa et al., 2021). The AMPAR density measured via positron emission tomography (PET) with [^11^C]K-2 was consistent with the local AMPAR protein distribution in surgical specimens of patients with mesial temporal lobe epilepsy (Miyazaki et al., 2020). Moreover, a recent PET-functional magnetic resonance imaging (fMRI) multimodal imaging study revealed a strong correlation between AMPAR density measured via [^11^C]K-2 PET and functional connectivity density in the human brain, underscoring the pivotal role of [^11^C]K-2 PET as a synaptic biomarker (Yatomi et al., 2024). This imaging technique has enabled the visualization and comparison of AMPAR distribution patterns across the entire brain in psychiatric diseases. Notably, BD shows widespread alterations relative to healthy controls (HCs), whereas MDD does not, and the relationship between regional AMPAR density and symptom severity diverges between the two disorders (Hatano et al., 2024).

Despite these prior AMPA-PET studies in psychiatric disorders, the clinical application of AMPAR imaging remains constrained by inter-subject heterogeneity in AMPAR density and the limitations of traditional group-level comparisons. In our earlier work, significant group-mean differences in regional AMPAR density were detected between diagnostic categories, yet the individual SUVR distributions of BD, MDD, and healthy controls overlapped substantially, illustrating that a single threshold based on mean shifts is diagnostically inadequate (Hatano et al., 2024). Such overlap arises because first-level, region-wise comparisons ignore the complex spatial covariance among brain areas. Consequently, a classification framework that exploits whole-brain pattern information and inter-regional interconnectivity is required. ML and multivariate pattern-analysis techniques have emerged as powerful tools for psychiatry because they can uncover subtle, multivariate patterns that elude conventional univariate tests (Chekroud et al., 2021; Lee et al., 2021; Ray et al., 2022). Neuro-imaging work illustrates this advantage: Redlich et al. (2014) discriminated BD from unipolar depression with 76% accuracy using support-vector machines on structural MRI despite minimal ROI-level mean differences; Winter et al. (2024) showed that multivariate models outperformed any single metric when group effects were as small as η^2^ < 0.02.

Inspired by these findings, we used a whole-brain partial least squares (PLS) classifier on [^11^C]K-2 PET maps to test whether multivariate AMPAR patterns can reliably distinguish BD from MDD during depressive episodes, thereby providing an objective biomarker for early differential diagnosis. Because the clinical challenge lies in separating depressive BD from MDD rather than manic BD, our analysis was restricted to patients in the depressive state.

## Materials and methods

### Ethics statement

This study comprised two clinical studies registered under the following IDs: UMIN000025132, jRCTs031190150. The Yokohama City University Human Investigation Committee and the Yokohama City University Certified Institutional Review Board approved this study, following the Ethical guidelines for medical and health research involving human participants by the Japan Ministry of Health, Labor, and Welfare and the Clinical Trials Act in Japan. Both studies were conducted at the Yokohama City University Hospital, Keio University Hospital, Kyushu University Hospital, and University of Fukui Hospital between August 2016 and April 2022. All participants provided written informed consent after receiving detailed information on the study protocol. Decision-making capacity was assessed using the MacArthur Competence Assessment Tool for Clinical Research (Appelbaum and Grisso, 2001).

### Participants

The selection criteria for participants are detailed in the Supplementary Methods. The first study (UMIN000025132) included male inpatients and outpatients aged 30–49 years who met the DSM-IV criteria for BD or MDD, as assessed using the Structured Clinical Interview for DSM-IV (SCID-I)/DSM-IV. The second study (jRCTs031190150) included male and female patients aged 20–59 years to broaden generalizability. To specifically examine misclassification risk during depressive episodes, only patients in a depressive state were included. The depressive state was defined based on the criteria of the International Society for Bipolar Disorders Task Force: Montgomery–Asberg Depression Rating Scale (MADRS) ≥ 8 and Young Mania Rating Scale (YMRS) ≤ 7 (Tohen et al., 2009).

### PET and MRI analyses

Participants underwent a 60 min PET scan with [^11^C]K-2 and an MRI scan. Detailed imaging settings are provided in the Supplementary Methods. [^11^C]K-2 was synthesized locally at each study site following the Good Manufacturing Practice guidelines. PET images from Keio University Hospital were processed with a 5.0 mm full-width at half-maximum (FWHM) Gaussian filter to match the resolution of PET images from other sites. Summed images from 30 to 50 min after [^11^C]K-2 injection were obtained using Magia (Karjalainen et al., 2020) and normalized to whole brain radioactivity, resulting in standardized uptake value ratio (SUVR) images. Spatial normalization to MNI standard space was performed using DARTEL in SPM12, followed by smoothing with an 8 mm FWHM Gaussian kernel.

### Statistical analysis

Demographic and clinical characteristics were compared between the BD and MDD groups using Pearson’s chi-squared test for categorical variables and Student’s *t*-test for continuous variables. SUVR values between patients with BD and those with MDD were compared using voxel-wise two-sample *t*-tests. A false discovery rate (FDR) correction at *P* < 0.05 (cluster-level, FDRc) was applied to control for multiple comparisons. A gray matter mask (voxels > 10% probability in SPM12 tissue maps) was applied to minimize false positives. For visualization, the FDR-corrected voxel mask was intersected with the Hammersmith atlas; mean SUVR was then extracted for each ROI, and individual values were displayed as scatter plots.

### Discriminant analysis using the partial least squares algorithm

**FIGURE 1:**
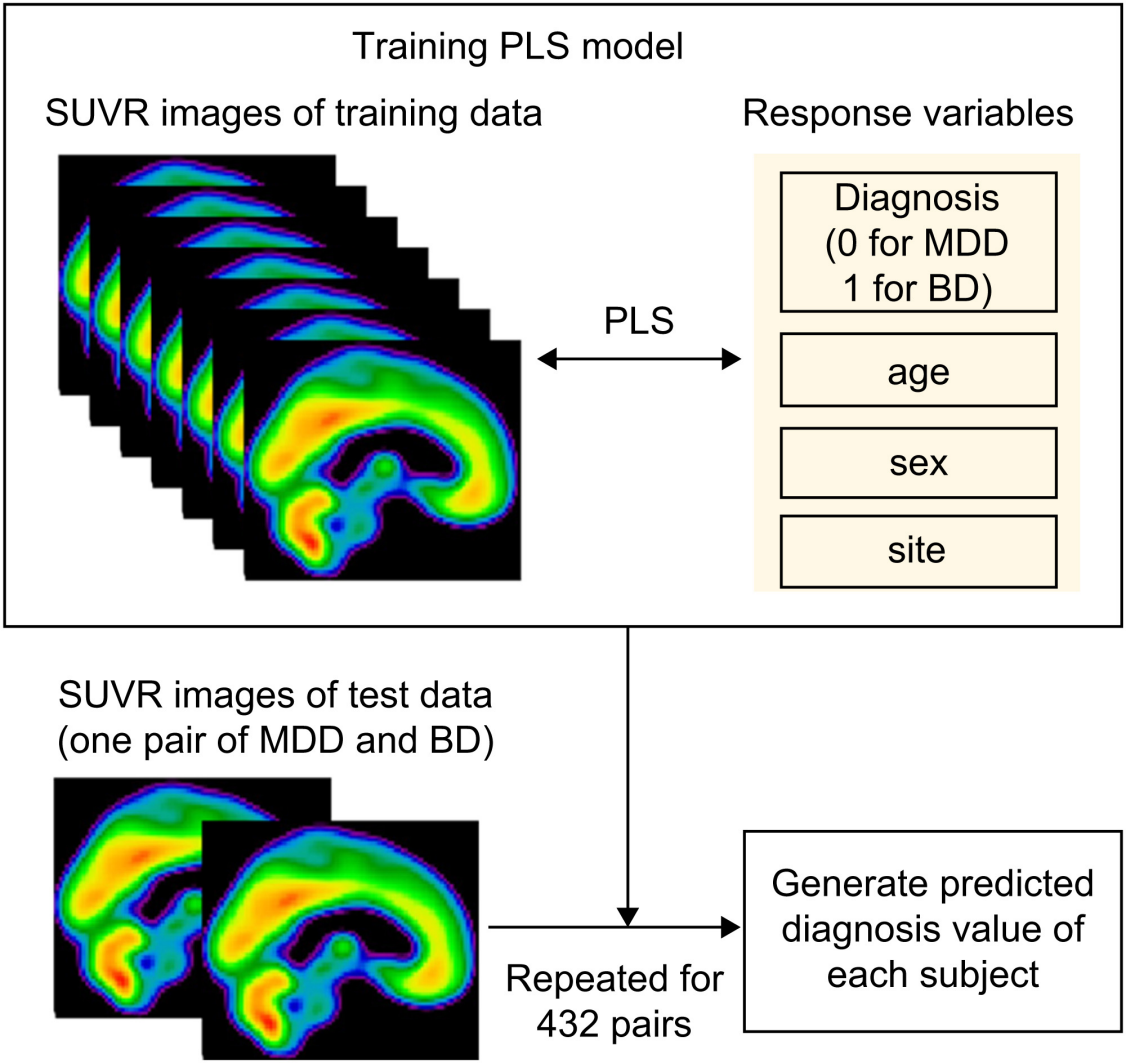
Schema of the partial least squares algorithm. To differentiate between BD and MDD, we employed a partial least squares (PLS) algorithm using standardized uptake value ratio (SUVR) values as explanatory variables and disease status (0 for MDD and 1 for BD), along with age, sex, and site as the response variables. Model performance was assessed using a leave-one-pair-out cross-validation approach, where each of the 16 patients with BD was paired with one of the 27 patients with MDD, generating 432 PLS models. For each iteration, the model was trained on the remaining data and tested on a single patient-control pair. Predicted values were generated for each test pair, yielding 27 predicted values per patient with BD and 16 predicted values per patient with MDD. The mean predicted value for each participant was calculated by averaging their respective predicted values. MDD, major depressive disorder; BD, bipolar disorder; PLS, partial least squares.

Bipolar disorder was differentiated from MDD using a PLS algorithm, with SUVR values as explanatory variables (Figure 1). Voxel-wise analysis was performed across the whole brain, but we applied a mask that included only voxels with SUVR values > 0.9 across all participants. This mask excluded voxels with low PET signal and was slightly more restrictive than a standard gray matter mask (gray matter probability > 10%), allowing us to focus on reliable high-signal regions. This reduced the number of explanatory variables. Response variables comprised disease status (0 for MDD and 1 for BD), along with age, sex, and site, to account for potential confounding effects of these covariates on disease classification. We selected partial least-squares (PLS) *a priori* and did not examine alternative classifiers for three reasons. First, even after masking, each scan contained ∼70 000 voxels whereas the cohort comprised only 43 participants; PLS projects these highly collinear predictors onto a small set of latent components that maximize covariance with the diagnostic label, thereby reducing dimensionality and limiting over-fitting. Second, unlike principal component analysis, PLS incorporates the outcome variable during dimensionality reduction, helping to mitigate potential confounding given the pleiotropic roles of AMPAR in the central nervous system. Third, the resulting voxel-wise β-weights can be rendered as spatial weight maps, allowing direct neurobiological interpretation of each region’s contribution to classification. PLS regression was performed using 10 latent variables. The number of components was chosen because the cumulative explained-variance curve reached a clear plateau at 10 components (Supplementary Figure 1). Model performance was assessed using a leave-one-pair-out cross-validation approach, where each of the 16 patients with BD was paired with one of the 27 patients with MDD, generating 432 PLS models. For each iteration, the model was trained on the remaining data and tested on a single patient-control pair. We chose leave-one-pair-out cross-validation rather than the more common leave-one-out procedure because leave-one-out would exacerbate the class imbalance whenever the single held-out subject happened to be a BIP participant. Predicted values were generated for each test pair, yielding 27 predicted values per patient with BD and 16 predicted values per patient with MDD. The mean predicted value for each patient was calculated by averaging their respective predicted values. A receiver operating characteristic (ROC) curve was constructed using the values, and the area under the curve (AUC) was computed. The optimal threshold was determined using the Youden Index, which maximizes the sum of sensitivity and specificity. To visualize which voxel values the PLS model prioritized for disease classification, the beta coefficients corresponding to disease status were extracted from a representative model and visualized as an image. As sensitivity analysis, we repeated the PLS regression with duration of illness (DOI) added to the response block (disease status + age + sex + site + DOI) to evaluate the robustness of the classification to illness-duration differences.

## Results

### AMPAR profiles in patients with BD and MDD in a depressive state

**FIGURE 2:**
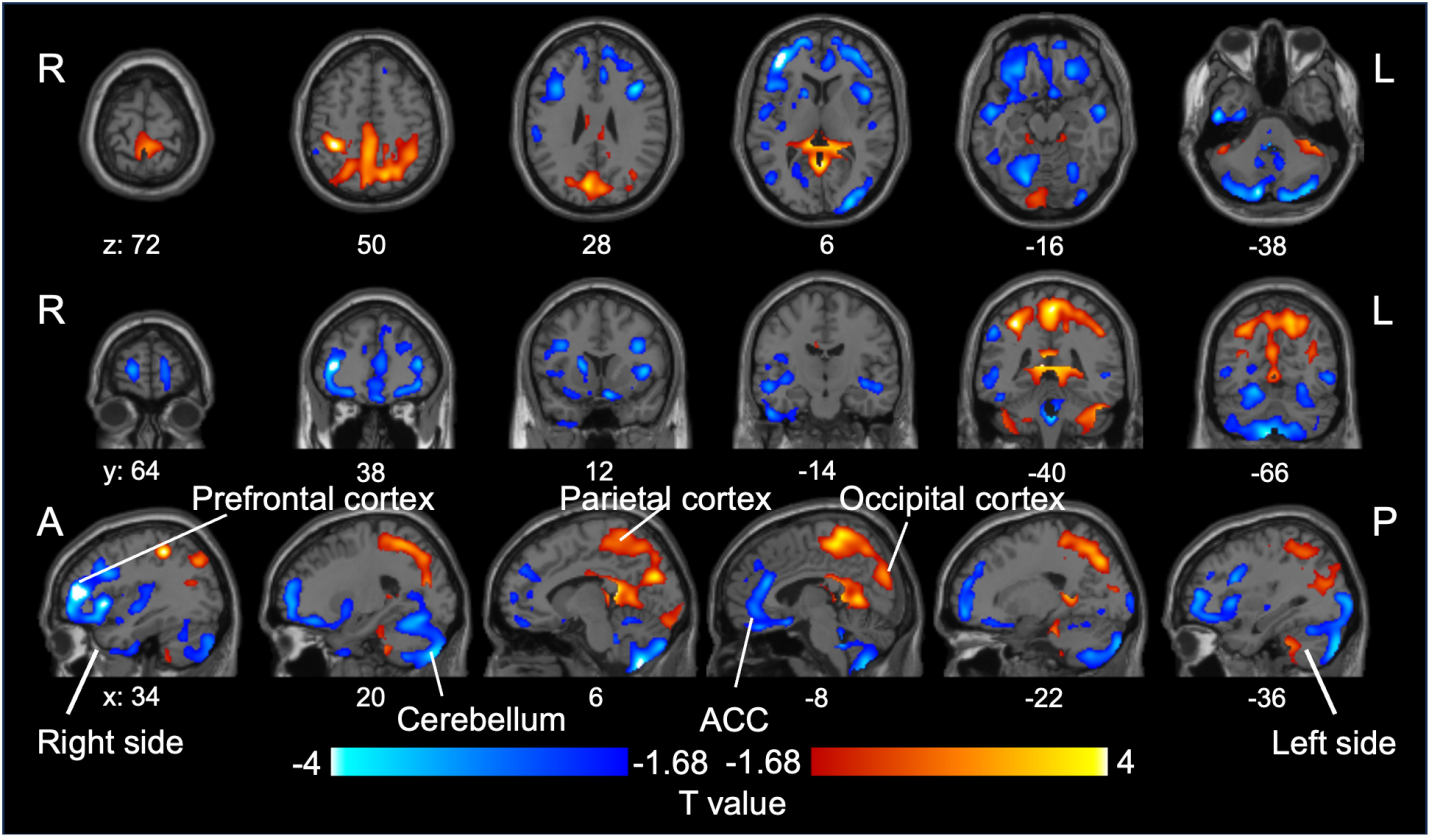
Group difference in SUVR values between BD and MDD. Relative reduction (blue) and increase (red) in [^11^C]K-2 retention in patients with BD compared to those with MDD (*P* < 0.05, increase in [^11^C]K-2 retention: T > 1.68, reduction in [^11^C]K-2 retention: T < –1.68, one-tailed, FDRc). MDD, major depressive disorder; BD, bipolar disorder; SUVR, standardized uptake value ratio.

In total, 16 and 27 patients with BD and MDD, respectively, met the inclusion criteria (Supplementary Figure 2). One participant with MDD met DSM-5 criteria for specific phobia, whereas no other participant met criteria for any current or lifetime psychiatric comorbidity, including anxiety disorders, personality disorders, or neurodevelopmental disorders. No significant differences were observed in age, sex, or severity of depressive symptoms between the two groups; however, the BD group had approximately twice the illness duration of the MDD group. Table 1 shows participants’ demographic and clinical characteristics. We provide detailed individual-level medication histories in Supplementary Tables 1, 2. Within the BD group, 15 of 16 patients were receiving lithium, often in combination with other agents. In the MDD group, medication classes overlapped substantially, and three patients were receiving lithium augmentation. On comparing SUVR values between patients with BD and MDD in a depressive state, we found that patients with BD had decreased SUVR values in the prefrontal cortex, including the dorsolateral prefrontal cortex, anterior cingulate cortex, and cerebellum, compared with those of patients with MDD. Conversely, patients with BD had increased SUVR values in the parietal and occipital cortices compared to those of patients with MDD (Figure 2). Supplementary Figure 3 presents individual scatter plots of SUVR values for each region that showed a significant group difference.

**TABLE 1:** Demographic and clinical characteristics of the participants.

Characteristics	MDD (N = 27)	BD (*N* = 16)	t-value/χ ^2^	*P*-value
Age, years	42.3 ± 7.7	42.0 ± 8.5	0.17	0.87
Female, *n* (%)	5 (33.3)	9 (56.3)	3.7	0.053
Duration of illness^a^	8.8 ± 8.1	16.6 ± 8.6	3.0	0.005
MADRS score	20.6 ± 8.8	16.6 ± 5.7	-1.6	0.10
YMRS score	NA	2.6 ± 2.5	–	–
Medication variable (*n*, %)	–	–	–	–
Lithium	3 (11.1)	15 (93.8)	33.1	< 0.001
Other mood stabilizer	0 (0)	4 (25.0)	5.9	0.015
Antidepressant	27 (100)	2 (12.5)	39.5	< 0.001
Antipsychotic augmentation	8 (29.6)	5 (31.3)	0.01	0.92

*^a^P* < 0.05. MDD, major depressive disorder; BD, bipolar disorder; MADRS, Montgomery Åsberg Depression Rating Scale; YMRS, Young Mania Rating Scale; NA, not applicable.

### Cell surface AMPAR distribution predicts BD or MDD diagnosis

**FIGURE 3:**
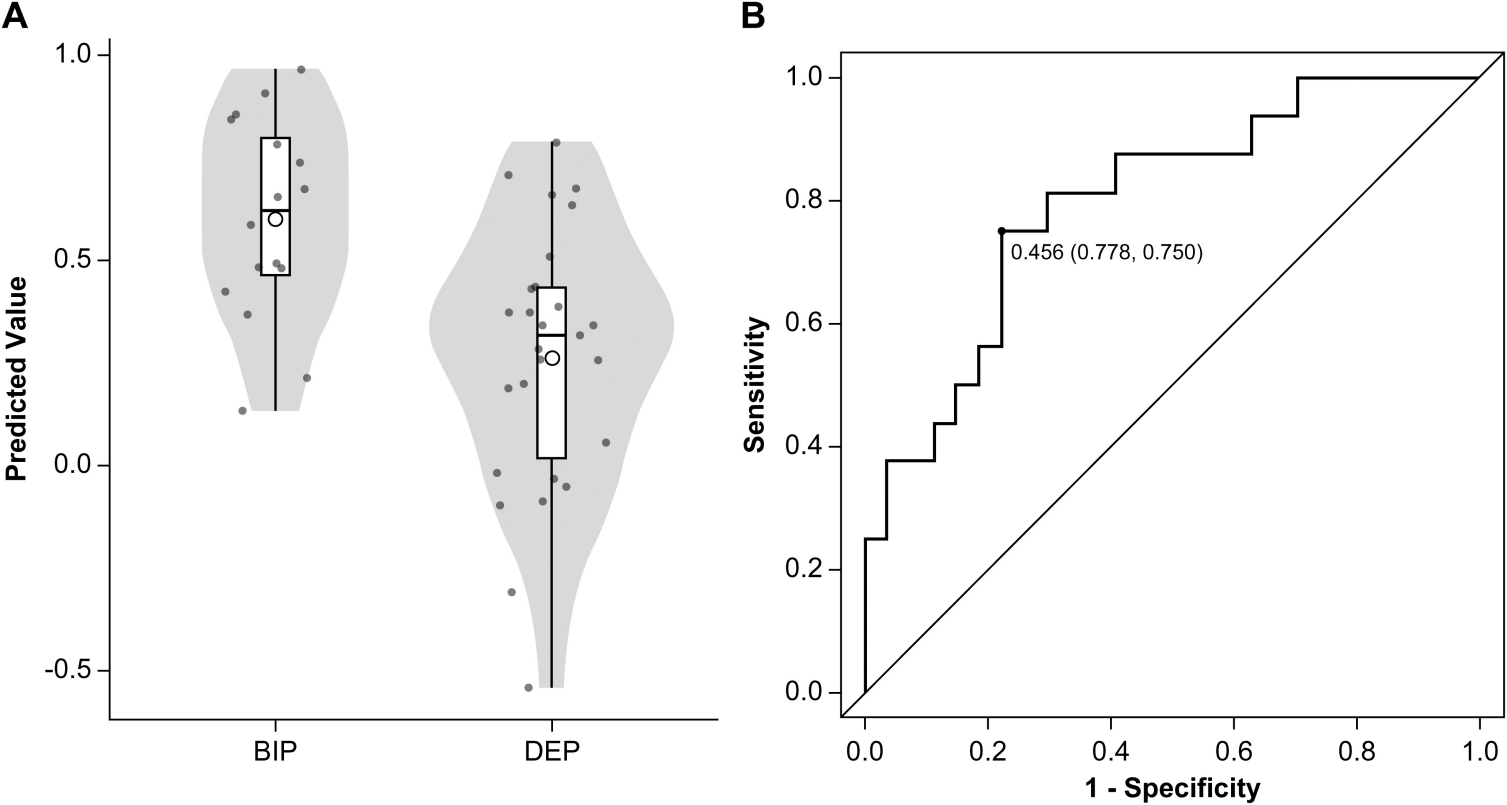
Classification performance of the partial least squares algorithm. **(A)** Predicted values from the PLS models are displayed using a violin plot and box plot. The means of the predicted values are represented by circular points. **(B)** Receiver operating characteristic curve (solid line) illustrates the classification performance of the PLS algorithm in distinguishing patients with BD from those with MDD. The area under the curve was 0.80 (95% confidence interval: 0.66–0.94). The optimal cutoff point was 0.456, providing a sensitivity of 75.0% for identifying BD (12 of 16 patients), a specificity of 77.8% (21 of 27), a positive predictive value of 66.7% (12 of 18), and a negative predictive value of 84.0% (21 of 25). MDD, major depressive disorder; BD, bipolar disorder; PLS, partial least squares.

**FIGURE 4:**
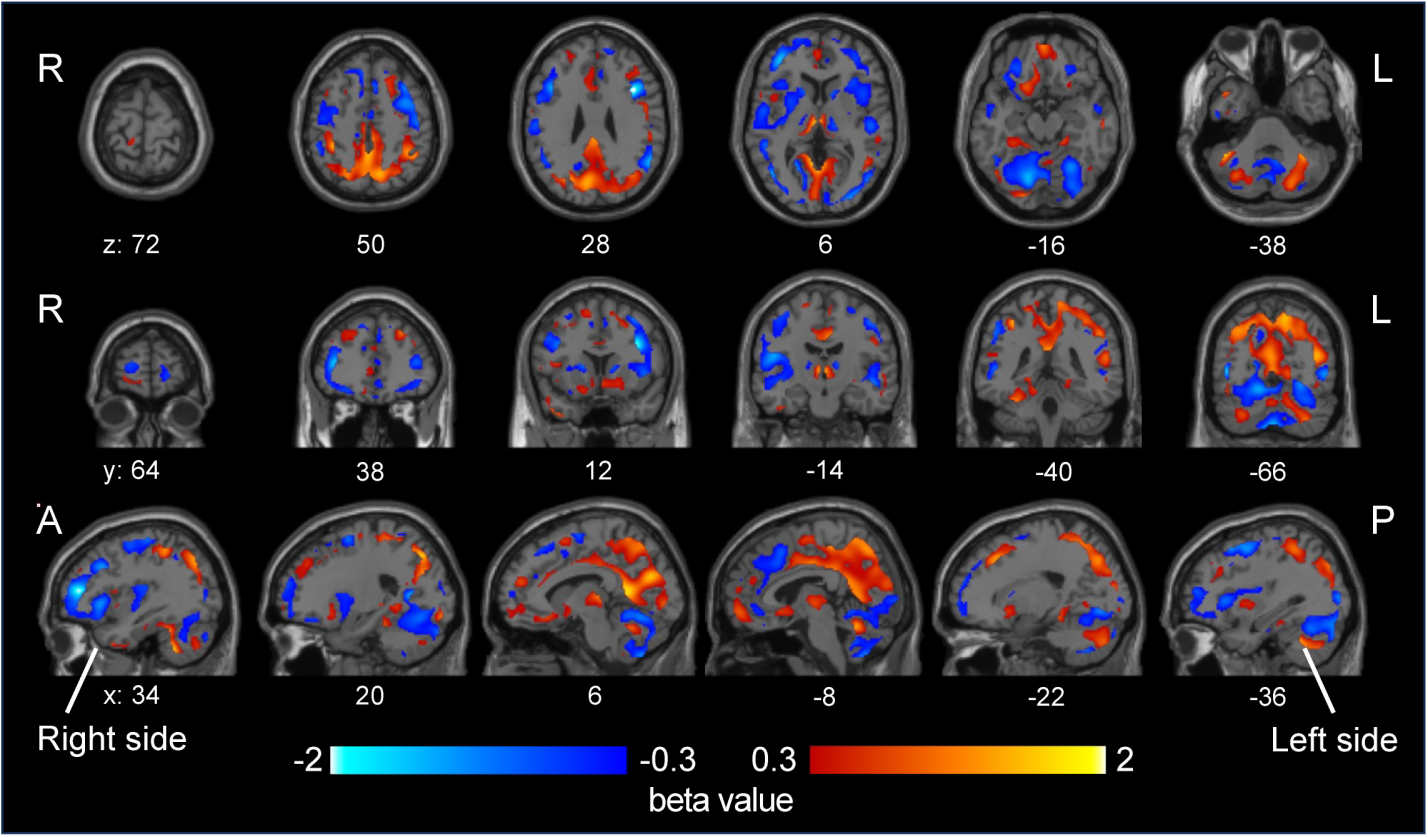
Beta coefficients of disease status from a representative partial least squares model. The beta coefficients from a representative partial least squares model, which are associated with disease status, are shown in the visualization. A higher beta coefficient (red) indicates that the predicted value increases as the SUVR value of a corresponding voxel increases. As a higher predicted value suggests a stronger likelihood of BD diagnosis, these coefficients highlight the relationship between specific voxel SUVR values and the probability of distinguishing BD from MDD. The opposite is true for negative beta values (blue). MDD, major depressive disorder; BD, bipolar disorder; SUVR, standardized uptake value ratio.

Partial least-squares algorithms calculated predicted classification scores for each patient, with values closer to 0 indicating a stronger resemblance to MDD and values closer to 1 suggesting a higher likelihood of BD. A scatter plot was constructed to illustrate the distribution of these predicted values between BD and MDD (Figure 3A). ROC analysis of these predicted values yielded an AUC of 0.80 (95% CI: 0.66–0.94) (Figure 3B). The optimal threshold, determined using the Youden Index, was 0.456, resulting in a sensitivity of 75.0%, specificity of 77.8%, PPV of 66.7%, and NPV of 84.0%. According to the beta coefficients in a representative PLS model, decreased SUVR values in the prefrontal cortex, anterior cingulate, and cerebellum and increased SUVR values in the occipital lobe, parietal lobe, posterior cingulate gyrus were key contributors to distinguishing BD from MDD (Figure 4). When DOI was included as an additional response variable, the classification performance remained stable (AUC = 0.81, 95% CI 0.67–0.94; Supplementary Figure 4), confirming that illness-duration differences did not account for the main findings.

## Discussion

In this study, we investigated whether *in vivo* measurements of AMPAR density help differentiate BD from MDD during depressive episodes. By leveraging the novel PET tracer [^11^C]K-2, we observed distinct spatial patterns of AMPAR distribution in BD compared to those in MDD and notably lower SUVR values in the prefrontal and anterior cingulate cortices and cerebellum but higher SUVR values in the parietal and occipital cortices. Using a PLS classification algorithm, we achieved clinically meaningful discrimination between BD and MDD (AUC = 0.80). These results suggest that quantifying cell surface AMPARs could enhance diagnostic precision in mood disorders, particularly when differentiating BD from MDD during depressive episodes.

### Main findings

Our study demonstrates that BD and MDD exhibit distinct AMPAR distribution patterns during depressive episodes. BD patients showed lower SUVR in the prefrontal cortex, anterior cingulate cortex, and cerebellum, but higher SUVR in the parietal and occipital cortices relative to MDD. Importantly, PLS analysis confirmed that SUVR differences in these regions provided the strongest discriminatory power between the two disorders. Notably, this spatial pattern replicates our earlier BD-versus-HC findings (Hatano et al., 2024): BD again exhibited lower frontal/ACC and higher parietal/occipital SUVR, whereas MDD did not differ from controls. The convergence suggests that AMPAR alterations in BD are trait-like and persist irrespective of mood state, whereas MDD shows mainly state-dependent changes. Reduced AMPAR availability in the prefrontal cortex and cerebellum may indicate impaired top-down emotion regulation. The dorsolateral and ventromedial PFC are central to cognitive control and affect regulation, while posterior cerebellar regions (Crus I/II) contribute to affective processing through their connectivity with limbic and prefrontal areas (Schmahmann and Sherman, 1998; Turner et al., 2007; Phillips et al., 2008; Etkin et al., 2011; Adamaszek et al., 2017). Structural and functional abnormalities in posterior cerebellar regions—including lobule VII (vermis VII and hemispheric Crus I/II)—have been repeatedly associated with mood lability in bipolar disorder (Mills et al., 2005; Phillips et al., 2015; Chen et al., 2019). Conversely, elevated AMPAR density in the parietal and occipital cortices suggests hyper-excitability of posterior sensory–attentional networks responsible for sensory integration, visuospatial representation, and attentional allocation, and aberrant parietal–salience connectivity has been reported in bipolar depression (Hibar et al., 2018; Yu Z. et al., 2020; Han et al., 2025). Collectively, BD appears to involve broad dysregulation across both regulatory (frontal–cerebellar) and sensory–attentional (parietal/occipital) circuits, whereas AMPAR alterations in MDD are more localized and state dependent. These trait-like AMPAR patterns strengthen the case for AMPAR PET imaging as a biomarker for identifying enduring pathophysiological features of BD. Future longitudinal studies spanning multiple mood states are required to determine whether these differences represent stable trait markers or fluctuate with clinical presentation.

Given the clinical importance of early and accurate BD identification—particularly to avoid antidepressant monotherapy, which can precipitate manic episodes—neurobiological markers have been explored to distinguish BD from MDD during depressive status. These include various neuroimaging (e.g., cortical morphology, resting-state connectivity) and blood-based (e.g., metabolomic or proteomic signatures) biomarkers. Regarding MRI research, many studies indicate that the salience and central executive networks, as measured via fMRI, can help discriminate between the two disorders, although the reported AUC values vary widely (Siegel-Ramsay et al., 2022). Additionally, several fMRI-based studies have reported better accuracy exceeding 80% (Almeida et al., 2013; Grotegerd et al., 2013; Yu H. et al., 2020; Xi et al., 2023), although these models have not been prospectively validated in larger trials. Supervised ML approaches have recently been employed to classify participants based on complex, multivariate brain imaging data. Several studies have applied ML to differentiate BD from MDD; however, the results vary depending on the modality. Tomasik et al. (2024) used extreme gradient boosting on blood-based metabolomic data to identify BD cases misdiagnosed as MDD among individuals who had received an MDD diagnosis within the previous 5 years, yielding AUCs of approximately 0.71–0.73. In contrast, Salvetat et al. (2024) integrated multiple RNA-editing–based gene markers and employed the extremely randomized trees algorithm, achieving an AUC of approximately 0.9 when classifying patients with a depressive episode as BD or MDD. Our study is the first to differentiate BD from MDD using AMPAR density measured via [^11^C]K-2 PET. Our PLS-based model reached an AUC of 0.80, indicating good accuracy. Additionally, the PLS model achieved sensitivity and specificity of 75.0% and 77.8%, respectively, at a threshold determined by the Youden Index. These metrics indicate a reasonable balance between capturing bipolar cases and minimizing false positives. Thus, our model represents a promising avenue for objectively distinguishing BD from MDD.

### Strengths and limitations

A major strength of this study is its novelty in distinguishing BD from MDD using an ML approach based on postsynaptic AMPAR density, an indicator directly linked to glutamatergic synaptic function. Some imaging and peripheral biomarker studies have attempted similar classifications, but AMPARs, given their fundamental role in excitatory neurotransmission, likely represent a more pathophysiologically direct target. Moreover, because neuromodulation or pharmaceuticals could alter postsynaptic AMPAR density, this biomarker could be manipulated therapeutically. Thus, AMPA-PET-based diagnosis may directly facilitate treatments targeting glutamatergic signaling. Unlike many existing clinical methods, this approach offers mechanistic insights that can be explored further in preclinical animal models. Experimentally manipulating AMPAR expression in key brain regions (e.g., frontal cortex and cerebellum) of animals could reveal causal links among AMPAR density, circuit-specific pathophysiology, and mood disorder symptoms. Another advantage lies in our specific recruitment of patients in a depressive state, reflecting the most challenging real-world scenario for differentiating BD from MDD. AMPAR distribution may vary among mood states; however, focusing on individuals currently experiencing depressive symptoms likely enhanced classification accuracy by reducing confounding from other mood phases.

Nonetheless, some limitations must be acknowledged. First, our sample size was relatively small, and the groups were imbalanced. We used a robust PLS model with leave-one-pair-out cross-validation to mitigate this issue; however, because no external validation cohort was available, this approach provides only an internal estimate of performance. Larger, fully independent samples are needed to verify generalizability before any clinical application. Second, we were unable to recruit treatment-naïve patients at very early disease stages because definitive diagnostic labels are difficult to assign without long-term clinical follow-up. In our sample, lithium was prescribed to 15 of 16 BD patients, and medication regimens in the MDD group overlapped substantially, limiting the variability needed to evaluate drug-specific effects on AMPAR density. Consequently, medication effects on AMPAR density could not be disentangled in the present study, and treatment-naïve—or at least lithium-naïve—cohorts will be essential for future validation. Third, although our MDD cohort was carefully evaluated by multiple psychiatrists, there is a possibility that a small subset of individuals could later experience a manic or hypomanic episode. Fourth, the BD group had a longer mean duration of illness than did the MDD group. Although DOI adjustment produced virtually identical results, larger cohorts with balanced illness durations will be necessary for definitive confirmation. Fifth, this multi-site study broadened recruitment but also introduced site-to-site differences. We accounted for potential site effects in the PLS model but cannot fully exclude residual variability. Finally, a practical limitation concerns radiotracer availability. Because [^11^C]K-2 has a physical half-life of only 20 min, it must be synthesized in an on-site cyclotron and radiochemistry facility, a resource that few hospitals possess. This requirement currently restricts AMPA-PET to major research centers and hampers early-stage clinical deployment. Low-cost, high-throughput blood-based biomarkers—some already achieving > 80% accuracy for BD vs MDD and entering clinical use in Europe (e.g., RNA-editing panels)—could serve as an initial screen (Salvetat et al., 2022, 2024; Hayashi et al., 2023). We envisage a two-tier pathway in which these peripheral tests flag high-risk cases, while AMPAR-PET provides circuit-level confirmation and phenotyping.

### Future directions

Building on the current findings, future research should employ larger, multicenter clinical trials to ascertain whether our PLS model can reliably distinguish BD from MDD across diverse populations and clinical settings. The diagnostic model can be refined and updated as more AMPA-PET data become available. Moreover, our group recently synthesized an [^18^F]-labeled derivative, [^18^F]K-40, which provides comparable measures of AMPAR distribution (Arisawa et al., 2022). A first-in-human study has now shown that [^18^F]K-40 reproduces the cerebral distribution and binding parameters of [^11^C]K-2 (Ichijo et al., 2025). The 110-min physical half-life of ^18^F supports centralized production and same-day delivery to surrounding PET facilities. Large multicenter cohorts scanned with [^18^F]K-40 will allow us to refine and externally validate the present classification model while mitigating the current tracer-availability barrier.

## Conclusion

Our results indicate that *in vivo* AMPAR imaging with [^11^C]K-2, combined with advanced multivariate approaches such as PLS, can accurately distinguish BD from MDD during depressive states. These findings highlight glutamatergic biomarkers as promising tools for improving classification precision, particularly when differentiating unipolar from bipolar depression remains clinically challenging. Although confirmation in larger and more diverse cohorts is necessary, our study underscores the potential of AMPAR imaging for mechanism-based diagnoses and optimized treatment strategies in mood disorders.

## Data Availability

The data analyzed in this study is subject to the following licenses/restrictions: all requests for raw and analyzed data and codes used in this study are promptly reviewed by the Yokohama City University Research Promotion Department to determine whether the request is subject to any intellectual property or confidentiality obligations and further inspected by the Institutional Review Board of Yokohama City University Hospital. Upon these approvals, derived data will be released via a material transfer agreement from the corresponding author. Requests to access these datasets should be directed to TT, takahast@yokohama-cu.ac.jp.
