# Supplementary material for: Differentiation between bipolar disorder and major depressive disorder based on AMPA receptor distribution
Source: Front Neural Circuits. 2025 Aug 4;19:1624179. doi: 10.3389/fncir.2025.1624179 (PMC12358400; doi:10.3389/fncir.2025.1624179)
Supplement: Supplementary file 1 [file Data_Sheet_1.docx]

**Supplementary Information**

**Supplementary Methods**

**In- and exclusion criteria**

**Patients with bipolar disorder.** In the first study (UMIN000025132) the inclusion criteria were male in- and outpatients 30–49 years of age who were capable of providing informed consent; and patients who were diagnosed with bipolar disorder according to the DSM-IV ^1^, using the SCID-I/DSM-IV ^2^, the DSM-5 ^3^, and the ICD-10 ^4^. In the second study (jRCTs031190150), the inclusion criteria were the same as those in the first study, other than the age range (i.e., 20-59 years) and sex (i.e., both men and women were included). To specifically examine misclassification risk during depressive episodes, only patients in a depressive state were included. The depressive state was defined based on the criteria from the International Society for Bipolar Disorders Task Force: Montgomery–Asberg Depression Rating Scale ≧ 8 and Young Mania Rating Scale ≦ 7. Exclusion criteria were the same for both studies; participants were excluded if they were pregnant, nursing, or desired to become pregnant; had a history of epilepsy; met criteria for substance abuse within six months of the study; had a positive urine drug screen for illicit drugs; received treatment with perampanel; met contraindications for MRI scan; had significant neurological or general medical conditions; or had abnormal laboratory test values of serum creatinine ≥ 1.5 mg/dl, AST ≥ 150 IU/L, or ALT ≥ 150 IU/L.

**Patients with depression.** In the first study (UMIN000025132) the inclusion criteria were: male in- and outpatients 30–49 years of age who were capable of providing informed consent; and patients who were diagnosed with major depressive disorder according to the DSM-IV ^1^, using the SCID-I/DSM-IV ^2^, the DSM-5 ^3^, and the ICD-10 ^4^. In the second study (jRCTs031190150), the inclusion criteria were the same as those in the first study, other than the age range (i.e., 20-59 years) and sex (i.e., both men and women were included). To specifically examine misclassification risk during depressive episodes, only patients in a depressive state (Montgomery–Asberg Depression Rating Scale ≧ 8) were included. Exclusion criteria were the same for both studies; participants were excluded if they were pregnant, nursing, or desired to become pregnant; had a history of epilepsy; met the substance abuse criteria within six months of the study; had a positive urine drug screen for illicit drugs; received treatment with perampanel; met contraindications for MRI scan; had significant neurological or general medical conditions; or had abnormal laboratory test values of serum creatinine ≥ 1.5 mg/dl, AST ≥ 150 IU/L, or ALT ≥ 150 IU/L.

**Settings and procedures of positron emission tomography (PET) and magnetic resonance imaging (MRI)**

**Settings.** The participants underwent a PET scan with [^11^C]K-2 and an MRI scan. [^11^C]K-2 was synthesized locally at each site in accordance with GMP ordinance. Injected dose of [^11^C]K-2 was 376.2 ±11.7 MBq (patients with bipolar disorder) and 368.7 ± 14.0 MBq (patients with depression).

*Yokohama City University Hospital.* PET imaging was performed with a TOSHIBA Aquiduo scanner (TOSHIBA Medical) and Celesteion PCA-9000A/2A (Canon medical). Aquiduo provided an axial FOV of 240 mm, and 80 contiguous 2.0 mm thick slices. A 4.7 s transmission scan was performed for attenuation correction (AC), then a 60 s intravenous injection of [^11^C]K-2 was given, which was followed by an emission scan of 60 min in all studies, with frames of 18 × 10 s, 2 × 30 s, 7 × 60 s, 1 × 2 min, 1 × 3 min, 3 × 5 min and 3 × 10 min. Dynamic images were reconstructed with a 2D-OSEM using 4 iterations, 14 subsets, a 128 matrix, a zoom of 2.8 and a 5.0 mm Gaussian filter. Celesteion provided an axial FOV of 240 mm and 96 contiguous 2.0 mm-thick slices. A 15.2 s transmission scan was performed for AC, and a 60 s intravenous injection of [^11^C]K-2 was administered, followed by an emission scan of 60 min, with 35 frames. Dynamic images were reconstructed with 3D-OSEM + TOF using 2 iterations, 20 subsets, a 128 matrix, a zoom of 1.0, and a 5.0-mm Gaussian filter. To permit accurate delineation of the brain regions for data analysis, each participant underwent an MRI scan on a GE DISCOVERY MR750 (General Electric Medical Systems). High resolution 3D-T1-weighted images (T1WI) were acquired using the following parameters: voxel size = 0.9 × 0.9 × 0.9 mm, repetition time (TR) / time to echo (TE) = 7.0/3.1 ms, flip angle (FA) = 8°, FOV = 220 mm, Matrix = 256 × 256.

*Keio University Hospital.* PET imaging was performed with a Biograph mCT Flow (Siemens Medical Solutions), which provided an axial FOV of 300 mm, and 111 contiguous 2.0 mm thick slices. A 6.56 s transmission scan was performed for AC, then a 60 s intravenous injection of [^11^C]K-2 was given, which was followed by an emission scan of 60 min, with 35 frames. Dynamic images were reconstructed with a 3D-OSEM + TOF using 4 iterations, 24 subsets, a 200 matrix, a zoom of 2.0 and a 2.0 mm Gaussian filter. To permit accurate delineation of the brain regions for data analysis, each participant underwent an MRI scan on a MAGNETOM Prisma (SIEMENS Healthineers) at university of Tokyo. High resolution 3D-T1WI were acquired using 3D MPRAGE protocol (voxel size = 0.8 × 0.8 × 0.8 mm, TR/TE = 2400/2.22 ms, FA = 8°, FOV = 208 mm, Matrix = 300 × 320).

*Kyushu University Hospital.* PET imaging was performed with a Biograph mCT Flow (Siemens Medical Solutions) and a Biograph Vision (Siemens Medical Solutions). Biograph mCT provided an axial FOV of 250 mm, and 165 contiguous 1.0 mm thick slices. A 9.38 s transmission scan was performed for AC, then a 60 s intravenous injection of [^11^C]K-2 was given, which was followed by an emission scan of 60 min, with 35 frames. Dynamic images were reconstructed with a 3D-OSEM + TOF using 5 iterations, 21 subsets and a 5.0 mm Gaussian filter. Biograph Vision provided an axial FOV of 357 mm, and 263 contiguous 1.0 mm thick slices. A 26.67 s transmission scan was performed for AC, then a 60 s intravenous injection of [^11^C]K-2 was given, which was followed by an emission scan of 60 min, with 35 frames. Dynamic images were reconstructed with a 3D-OSEM + TOF using 8 iterations, 5 subsets and a 5.0 mm Gaussian filter. To permit accurate delineation of the brain regions for data analysis, each participant underwent an MRI scan on an Ingenia 3.0-T scanner (Phillips). High resolution 3D-T1WI were acquired using 3D MPRAGE protocol (voxel size = 1.2 × 1.0 × 1.0 mm, TR/TE = 6.8/3.1 ms, FA = 9°, FOV = 170 mm, Matrix = 256 × 256).

*University of Fukui Hospital.* PET and MRI were simultaneously scanned with a Signa PET/MRI (GE Healthcare). Signa PET/MRI provided an axial FOV of 256 mm, and 89 contiguous 2.78 mm thick slices. For AC, a 3D radial MR acquisition for the zero-echo time (ZTE) method in the axial direction was performed with the following sequence: FOV 264 mm, matrix 110 × 110 × 116, voxel size 2.4 × 2.4 × 2.4 mm^3^, flip angle 0.8°, number of excitations 4, bandwidth ± 62.5 kHz and acquisition time of 41 s.　The ZTE-AC method was previously described.^5^ A 60 s intravenous injection of [^11^C] K-2 was performed and followed by an emission scan of 60 min, with 35 frames. Dynamic images were reconstructed with a 3D-OSEM + TOF using 3 iterations, 28 subsets, a 128 matrix, and a 3.0 mm Gaussian filter. High resolution 3D-T1WI and other MR images were acquired simultaneously using the following sequence: voxel size = 0.9 × 0.9 × 0.9 mm, TR/TE = 8.5/3.3 ms, FA = 8°, FOV = 196 mm, Matrix = 256 × 256).

**Supplementary Figure**

**Supplementary Figure 1. The cumulative explained-variance curve of PLS model.**


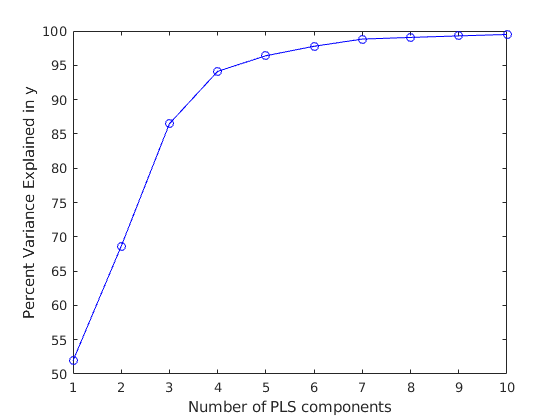


**Supplementary Figure 2**. **CONSORT Chart of BD and MDD
(a) CONSORT Chart of the first study**


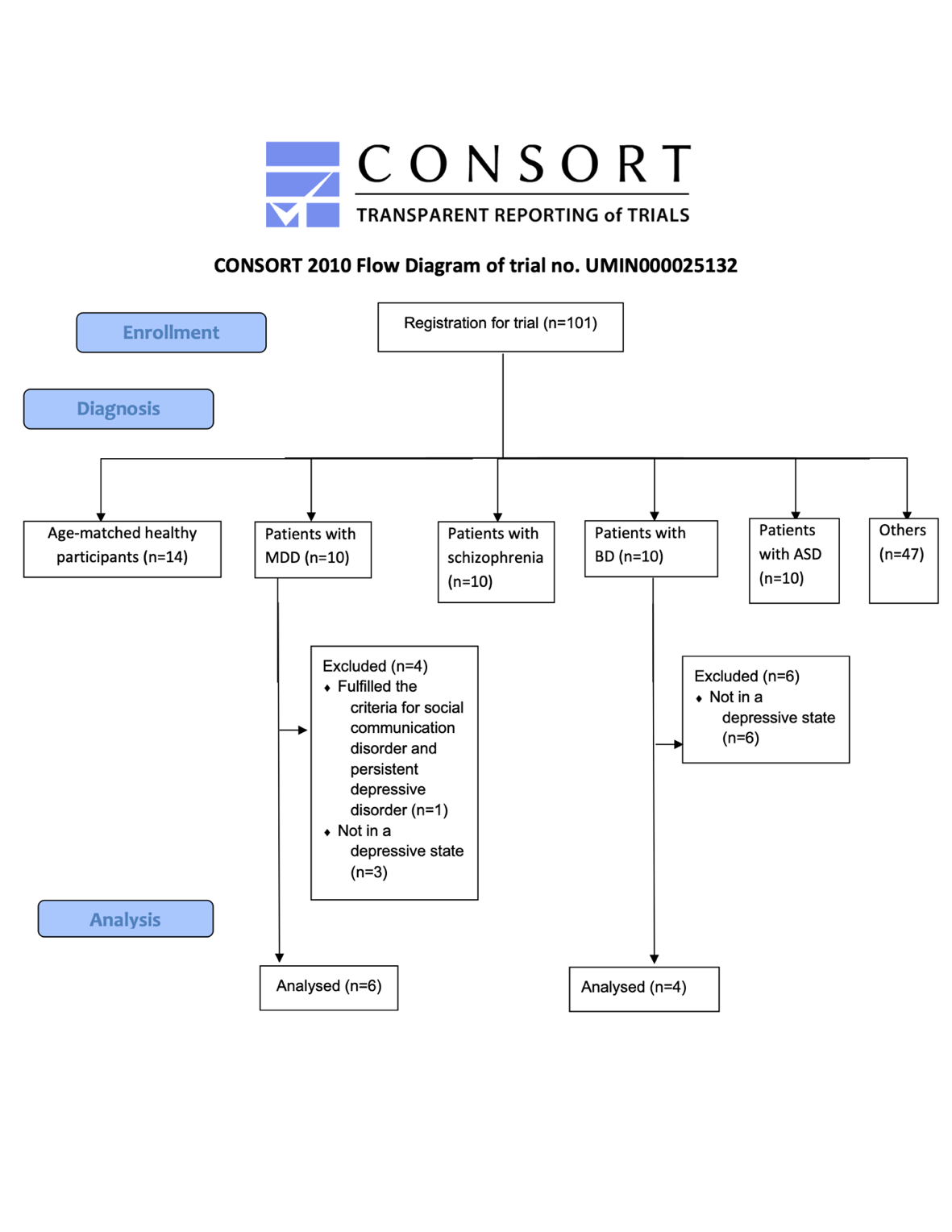


**(b) CONSORT Chart of the second study**


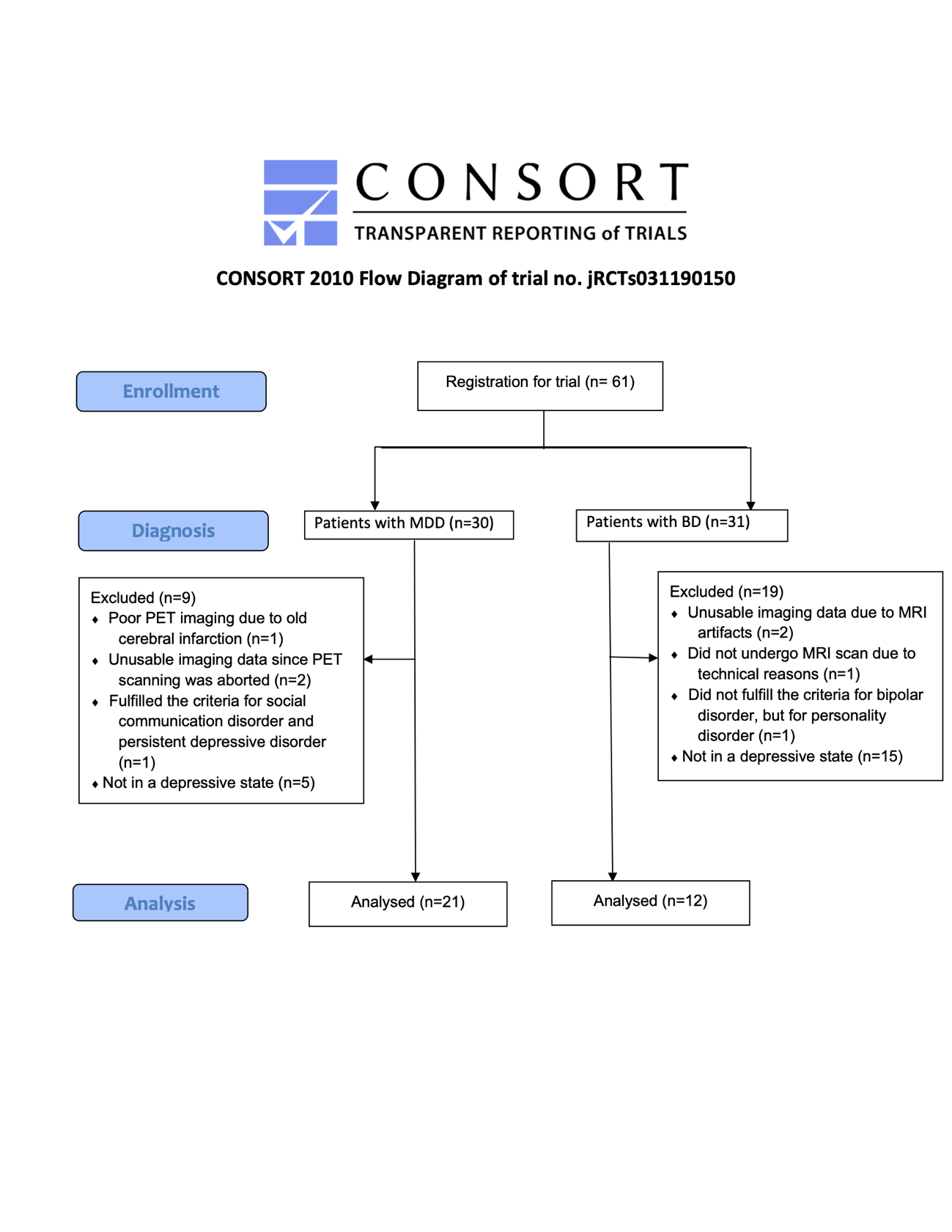


**Supplementary Figure 3.  Individual SUVR values in key brain regions.**

Scatter plots of [¹¹C]K-2 SUVR for BD and MDD in regions defined by the Hammersmith atlas: (A) prefrontal cortex, (B) parietal cortex, (C) occipital cortex, (D) anterior cingulate cortex, and (E) cerebellum. Each dot represents one participant; violin and box plots overlay the scatter to summarize the group-level distribution. SUVR values were extracted from ROI-wise analyses restricted to voxels that showed significant between-group differences in the whole-brain comparison (FDR-corrected P < 0.05).

**
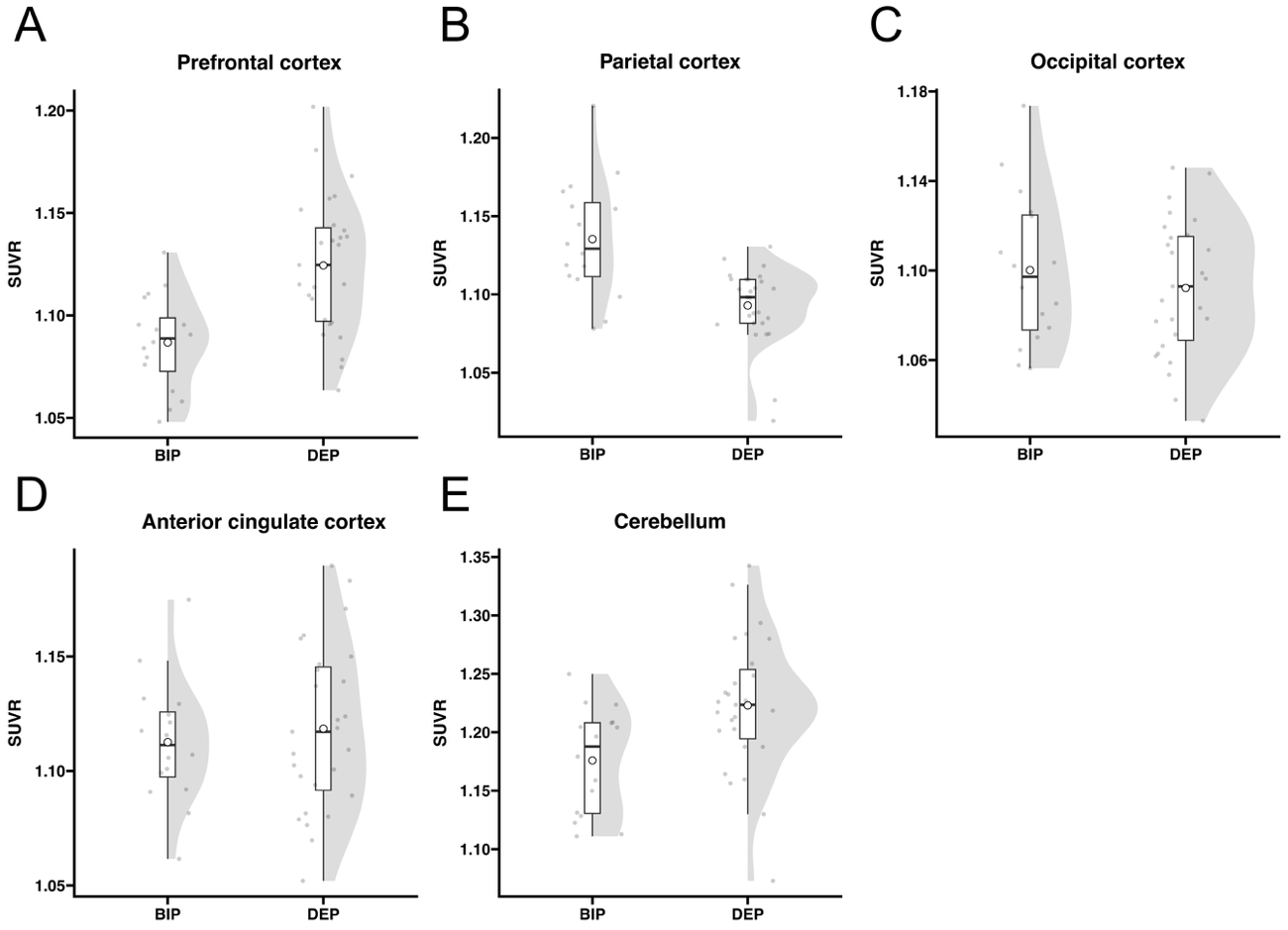
**

**Supplementary Figure 4. Diagnostic performance of the partial least squares algorithm with duration of illness as response variable.**

(a) Predicted values from the PLS models with duration of illness as response variables are shown as violin and box plots. The means of the predicted values are represented by circular points. (b) Receiver operating characteristic curve (solid line) illustrates the diagnostic performance of the PLS algorithm in distinguishing patients with BD from those with MDD. The area under the curve was 0.81 (95% confidence interval: 0.67–0.94). The optimal cutoff point was 0.382, providing a sensitivity of 87.5% for identifying BD (14 of 16 patients), a specificity of 66.6% (18 of 27), a positive predictive value of 60.9% (14 of 23), and a negative predictive value of 90.0% (18 of 20).

Abbreviations: MDD, major depressive disorder; BD, bipolar disorder; PLS, partial least squares

**
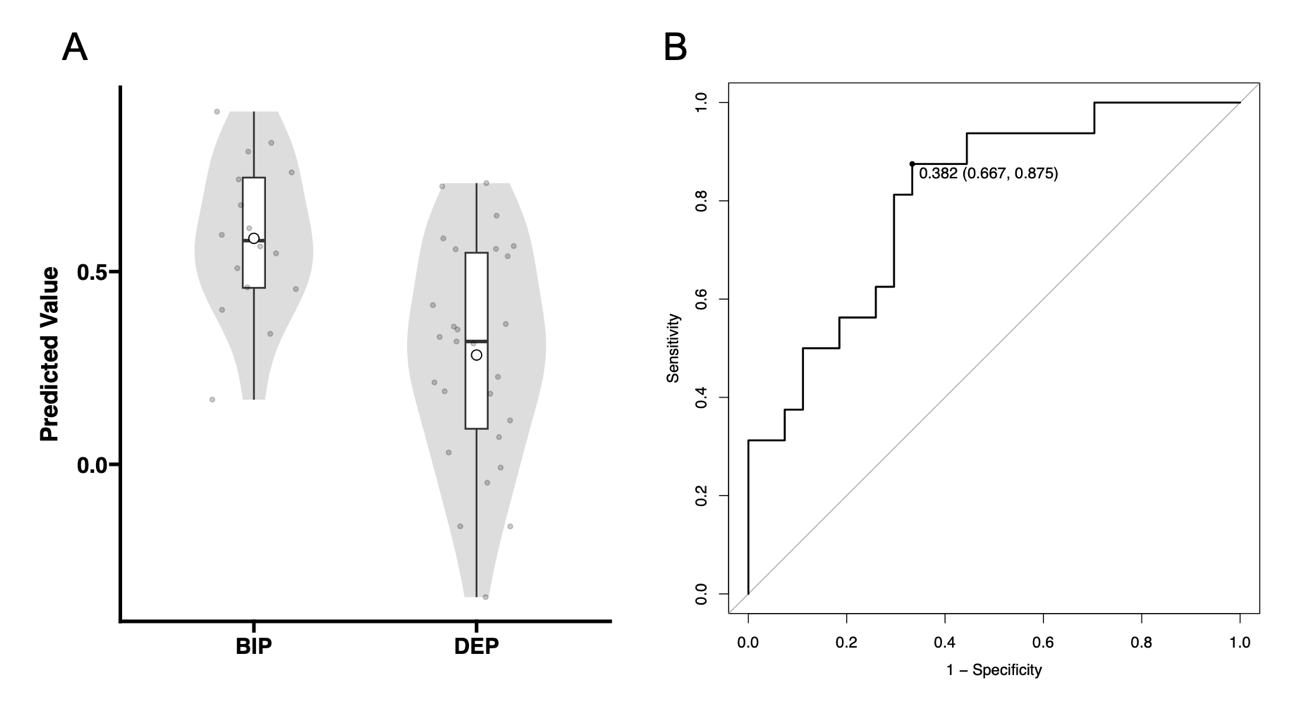
**

**Supplementary Table**

**Supplemental Table 1. Demographic and Clinical Characteristics of Patients with bipolar disorder.**

| No. | Age, years | Sex | DOI, years | MADRS total score | YMRS | Medications prescribed, mg/day, Psychotropic drugs are written in italics |
| --- | --- | --- | --- | --- | --- | --- |
| 1 | 38 | M | 11 | 11 | 5 | *lithium 600* |
| 2 | 35 | M | 10 | 16 | 2 | *lithium 1200* |
| 3 | 47 | M | 12 | 32 | 0 | *lithium 600, escitalopram 10, etizolam 0.5, quazepam 20*, polycarbophil calcium 1500 |
| 4 | 41 | M | 18 | 14 | 7 | *lithium 800, lamotrigine 200, aripiprazole 3, levomepromazine 10, suvorexant 20, ethyl loflazepate 1* |
| 5 | 39 | F | 19 | 20 | 0 | *lithium 600, trazodone 200, brotizolam 0.25* |
| 6 | 32 | M | 17 | 8 | 3 | *quetiapine 200, mirtazapine 15, clonazepam 1* |
| 7 | 29 | F | 10 | 22 | 4 | *lithium 900, aripiprazole 12, levocetirizine 10*, vonoprazan fumarate 10 |
| 8 | 43 | M | 26 | 14 | 4 | *lithium 600, lamotrigine 50* |
| 9 | 46 | F | 30 | 14 | 0 | *lithium 500* |
| 10 | 38 | F | 15 | 20 | 2 | *lithium 800, quetiapine 500, flunitrazepam 2, magnesium oxide 1000, lubiprostone 24, linaclotide 0.5* |
| 11 | 55 | F | 35 | 16 | 0 | *lithium 600, lamotrigine 200, lorazepam 1, flunitrazepam 2* |
| 12 | 46 | M | 6 | 14 | 7 | *lithium 800, zolpidem 10, levomepromazine 10, flunitrazepam 2* |
| 13 | 40 | F | 20 | 16 | 0 | *lithium 1300, methylphenidate hydrochloride 27* |
| 14 | 39 | F | 2 | 15 | 2 | *lithium 800, brexpiprazole 1, ramelteon 8, clonazepam 0.5*, clonidine 75, |
| 15 | 56 | F | 16 | 22 | 5 | *lithium 1000, clonazepam 1, brotizolam 0.25, lorazepam 1.5, promethazine 25* |
| 16 | 59 | F | 19 | 11 | 0 | *lithium 400, lamotrigine 100, zolpidem 10, clonazepam 1, suvorexant 20, sodium valproate 400, nitrazepam 5, levothyroxine sodium hydrate 75* |

M, male; F, female; DOI, duration of illness; HAMD-17, 17-item Hamilton Depression Rating Scale; YMRS, Young Mania Rating Scale

**Supplemental Table 2. Demographic and Clinical Characteristics of Patients with depression.**

| No. | Age, years | Sex | DOI, years | HAMD-17 total score | Medications prescribed, mg/day, Psychotropic drugs are written in italics |
| --- | --- | --- | --- | --- | --- |
| 1 | 44 | M | 7 | 15 | *sertraline 25* |
| 2 | 49 | M | 11 | 10 | *nortriptyline 100, loflazepate 1,* allopurinol 100 |
| 3 | 33 | M | 12 | 8 | *sertraline 100, nortriptyline 25, zolpidem 5, etizolam 1.5, alprazolam 1.2* |
| 4 | 39 | M | 7 | 10 | *sertraline 100, zolpidem 10* |
| 5 | 33 | M | 2 | 8 | *duloxetine 60, aripiprazole 3, quetiapine 75, lorazepam 1, brotizolam 0.25, suvorexant 20* |
| 6 | 48 | M | 7 | 9 | *flunitrazepam 1* |
| 7 | 37 | M | 3 | 6 | *escitalopram10* |
| 8 | 58 | M | 0 | 20 | *sertraline 100, eszopiclone 3* |
| 9 | 38 | F | 18 | 4 | *sertraline 100, aripiprazole 3* |
| 10 | 56 | M | 1 | 8 | *sertraline 50, lemborexant 10* |
| 11 | 41 | M | 0 | 25 | *duloxetine 60, mosapride citrate hydrate 5, eszopiclone 2* |
| 12 | 36 | M | 6 | 3 | *duloxetine 60, sertraline 100* |
| 13 | 26 | M | 4 | 11 | *fluvoxamine maleate 125* |
| 14 | 43 | M | 0 | 16 | *duloxetine 40, lemborexant 10, lorazepam 1.5* |
| 15 | 50 | F | 17 | 7 | *venlafaxine 112.5, quetiapine 225, sennoside 12* |
| 16 | 43 | M | 6 | 18 | *lorazepam 1.5, sertraline 75, zolpidem 5, loxoprofen 60* |
| 17 | 49 | F | 29 | 13 | *amoxapine 150, clomipramine 225, brexpiprazole 1, methylphenidate 18, zolpidem 10,  triazolam 0.5, levomepromazine 45,　clonazepam 2* |
| 18 | 45 | F | 21 | 6 | *vortioxetine 20, duloxetine 60, amoxapine 75, cloxazolam 1,　lamotrigine 325, aripiprazole 3*,  sennoside 36, magnesium oxide 1500, pitavastatin calcium 1 |
| 19 | 37 | M | 20 | 15 | *sertraline 75, zolpidem 5, suvorexant 20, clomipramine 30,  clomipramine 30, lithium 800, thyronamine 1.5, clonazepam 0.5* |
| 20 | 46 | F | 1 | 13 | *venlafaxine 150, mirtazapine 15, aripiprazole 6* |
| 21 | 42 | M | 7 | 25 | *amoxapine 30, zolpidem 10* |
| 22 | 47 | M | 17 | 22 | *aripiprazole 6, lorazepam 0.5, lithium 400, nortriptyline 70, zolpidem 10* |
| 23 | 33 | M | 7 | 17 | *brotizolam 0.25, flunitrazepam 1, trazodon 25, vortioxetine 20, suvorexant 20, venlafaxine 150* |
| 24 | 30 | F | 6 | 20 | *flunitrazepam 2, ramelteon 8, venlafaxine 225* |
| 25 | 49 | M | 2 | 23 | *brotizolam 0.25, quetiapine 25, paroxetine hydrochloride hydrate 50,  risperidone 2, eperisone 0.5, amitriptyline 75* |
| 26 | 43 | M | 24 | 13 | None |
| 27 | 46 | M | 1 | 8 | *duloxetine 60, bromazepam 12, lithium 200* |

M, male; F, female; DOI, duration of illness; HAMD-17, 17-item Hamilton Depression Rating Scale

**References.**

1. American Psychiatric Association. *Diagnostic and Statistical Manual of Mental Disorders, 4th ed.* American Psychiatric Association: Washington, D.C., 1994.

2. First MB, Spitzer RL, Gibbon M, Williams JBW. *Structured clinical interview for DSM-IV axis I disorders, clinician version (SCID-CV)*. American Psychiatric Press: Washington, D.C., 1996.

3. American Psychiatric Association. *Diagnostic and statistical manual of mental disorders (5th Ed)*. American Psychiatric Publishing: Arlington, VA, 2013.

4. World Health Organization. *The ICD-10 classification of mental and behavioural disorders: clinical descriptions and diagnostic guidelines*. WHO: Geneva, 1992.

5. Okazawa H, Ikawa M, Jung M, Maruyama R, Tsujikawa T, Mori T *et al.* Multimodal analysis using [(11)C]PiB-PET/MRI for functional evaluation of patients with Alzheimer's disease. *EJNMMI Res* 2020; **10**(1)**:** 30.
